# Barriers to finding and maintaining pet-inclusive affordable housing: Tenant experiences in Houston, Texas

**DOI:** 10.3389/fvets.2024.1465682

**Published:** 2024-10-28

**Authors:** Tess M. Mascitelli, Taryn M. Graham, Lauren Loney, Jennifer W. Applebaum, Christa M. Murray, Miranda Binns-Calvey, Sloane M. Hawes, Kevin Morris

**Affiliations:** ^1^Institute for Human-Animal Connection, Graduate School of Social Work, University of Denver, Denver, CO, United States; ^2^Independent Researcher, Toronto, ON, Canada; ^3^The Humane Society of the United States, Washington, DC, United States; ^4^Department of Environmental and Global Health, University of Florida, Gainesville, FL, United States

**Keywords:** pets, affordable housing, tenants, policy, housing insecurity

## Abstract

The city of Houston, Texas has a growing deficit of available and affordable rental units for low-income residents. Due to pet policies, the shortage of affordable housing potentially puts renters who own pets at greater risk of housing insecurity. In this qualitative study, we use a community-engaged approach to document the lived experiences of finding and maintaining affordable housing among 24 current, former, and aspiring pet owners. The majority of the participants identified as female, were aged 44–60 years, identified as Black, had a high school education, and were employed full-time or on disability or government assistance. Many expressed having experienced homelessness in the past and/or having lived in several different types of affordable housing over their lifetime. Participants highlighted challenges in finding pet-inclusive affordable housing, emphasized its importance, and discussed issues faced, such as high pet charges, size and breed restrictions, and confusion surrounding pet policies. Landlord relationships and living conditions varied, with safety concerns prevalent. Having one’s pet designated as an Emotional Support Animal made tenants feel safe and secure, knowing they could not be refused, evicted, or otherwise charged extra. Participants shared what is working well and what could be improved. This study concludes with recommendations for fair application and awareness of pet policies in affordable housing, drawing on participating tenants’ experiences and existing efforts for policy and practice improvements.

## 1 Introduction

Several studies have highlighted how lack of pet-inclusive housing contributes to housing insecurity of both people and pets (1). About 50–75% of rental housing allows pets in the U.S. (2, 3). However, 72% of renters state that pet-friendly housing is “hard to find” and it is estimated that only 8% of rental housing in the U.S. are truly “pet-inclusive” (defined as units without any restrictions on the breed, weight, or size of pets allowed on the property and without pet-related nonrefundable upfront fees or additional pet rents) (4). Renters with pets report feeling powerless, discriminated against, and insecure about the stability of their housing (5, 6). Many also report settling on lower quality housing, living in undesirable neighborhoods, and worrying about their ability to move due to overall lack of pet-inclusive units (5, 6). Pets may also be a barrier to exiting homelessness, with most pet owners experiencing homelessness reporting that they are not willing to abandon their pets in exchange for pet-prohibitive temporary shelter or housing (7). Lack of pet-inclusive housing also contributes to animal shelter overcrowding, as it is often cited as one of the main reasons why pets are relinquished to animal shelters (8, 9).

Marginalized populations may be disproportionately impacted by lack of pet-inclusive housing. Black, Indigenous, people of color (BIPOC) renters, for instance, are more likely to be lower-income renter households, due to wage and homeownership disparities relative to White households (10), and research has shown that rental housing in the U.S. that accepts pets is on average $200 USD per month more expensive than properties with “no pets allowed” policies (3). This is due to common practices by housing providers that include charging a pet deposit, additional pet fees, and/or pet rent (11). One study in the U.S. used geographic information systems (GIS) mapping to discover that predominantly White neighborhoods had significantly more pet-friendly rental properties available than predominantly Black neighborhoods (12). Similarly, an analysis of U.S. rental listings from the “Apartments.com” database revealed that rental units accepting pets have a higher financial burden in racially diverse communities compared to communities composed of primarily White residents (11). This policy deficit is not distinctive to the U.S. (1).

Individuals living in affordable housing may experience even greater disparities in availability and access to pet-inclusive housing. Affordable housing (also referred to as subsidized, public, or social housing) is defined as “housing in which the occupant is paying no more than 30 percent of gross income for housing costs, including utilities” (13). In many cities throughout the U.S. and internationally, there is a growing deficit of available and affordable rental units for low-income households overall (10). Only a limited number of studies have been conducted on pet policies specifically in affordable housing. For example, several studies have found that no-pet policies are common in housing for older adults and supportive housing for individuals with mental health challenges (14–17). An exploratory study conducted in Edmonton, Canada (18) found that decision-making about pet policies was inconsistent across affordable housing organizations, with policies informed by several factors, including the population of residents who live at the housing property, personal beliefs or experiences of staff at the agency, financial considerations, and the organization’s mission and vision. The lack of research on pet-inclusive affordable housing has prevented evidence-based policymaking efforts in this area (17, 18).

The goal of this present study is to address this gap in the literature by documenting affordable housing tenants’ lived experiences with finding and maintaining pet-inclusive affordable housing in Houston, Harris County, Texas, U.S.A. To our knowledge, this is the first paper to focus on the firsthand experiences of current, former, and aspiring pet owners primarily from underrepresented backgrounds (i.e., minoritized racial groups, low-income, low rates of employment, etc.) who are living in affordable housing units.

## 2 Methods

### 2.1 Qualitative approach

To explore the perspectives of pet owners living in affordable housing in Houston, TX, a phenomenological approach was adopted. Phenomenology is an approach that is used to explore the essence of a phenomenon from the perspectives of those with lived experience (19). Phenomenology allows for rich detail and nuanced narratives to help understand the phenomenon “as it is.” In doing so, new meanings and appreciations may be developed, with the goal to advance understanding of people’s realities and, in this paper, with the hopes of improving policy and practice.

### 2.2 Researcher characteristics and reflexivity

The identities and lived experiences of the authors have some similarities and notable differences from the research participants that are important to disclose. All authors are racially white individuals who hold advanced degrees. The majority of the authors are U.S. citizens, with one author who is a Canadian citizen. Most authors identify as cisgender women, with one author identifying as a cisgender man. All authors are pet owners who have personally experienced struggling to identify rental housing that was affordable and that allowed their pets. Some authors have personally experienced housing insecurity or have supported family members who were unhoused. However, none of the authors has resided in public or subsidized housing in the U.S. Some authors have been or are currently landlords who rent to tenants with pets. The authors’ professional experiences include: tenants rights and affordable housing law, housing authority leadership, social work with families impacted by the child welfare system, academic research and administration, and various roles within animal sheltering (e.g., community outreach, foster coordinator, volunteer). All authors acknowledge the influence their experiences and positionality have on their role in the research and met regularly to engage in collective reflexivity (20).

### 2.3 Setting and context

This study took place in Houston, TX, U.S.A. In 2020, when this study was designed, Houston was in the top 10 of metropolitan areas in the U.S. with the most severe shortages of affordable housing, with an estimated 19 available and affordable rental units per 100 extremely low-income households (10). Illustrative of this crisis, 46% of all Houston renter households are housing-cost burdened (21), meaning they spend greater than 30% of their monthly income on housing expenses. Further, Harris County had historic numbers of eviction filings in 2022 with Black families with children facing significantly higher eviction rates than other demographic groups (22). In 2021, almost 90,000 low-income renters were living in subsidized, affordable rental housing in Houston (23), with significant unmet needs in the community remaining: in January 2023, the Houston Housing Authority opened its public housing waitlist to a new group of applicants and within 1 month, more than 39,000 applications had been submitted (24). The Houston metro area ranks 4th and 8th in the country for dog and cat ownership, respectively, with more than half of all households reporting owning at least one dog or cat (25). While this study took place in the city of Houston, commodification of housing and ongoing patterns of racial and economic segregation in housing occurs all across the U.S. and in other countries (26, 27).

### 2.4 Sampling strategy

Multiple recruitment approaches were used. Fifteen local Houston community organizations sent recruitment emails and circulated flyers to their networks, including both human service providers and animal welfare organizations. Flyers were furthermore posted in four Houston Multi-Service centers and numerous Houston libraries. A recruitment poster was shared via Twitter and Facebook. Additionally, members of the research team and two representatives of the Houston Tenants Union engaged in door-to-door recruitment at properties managed by the Houston Housing Authority.

Interested participants were invited to contact the research team through email or phone. Eligibility criteria included: being at least 18 years old, looking for or living in affordable housing in Houston, and having previous, current, or desired future experiences with pet ownership (i.e., as a current, previous, or future pet owner). For this study, affordable housing was defined as any housing where tenants receive a financial subsidy to help pay rent or where rent was reduced for tenants earning below a certain income threshold. This included public housing properties, project-based Section 8 properties, Low Income Housing Tax Credit (LIHTC) properties, rental assistance programs, permanent supportive housing, transitional housing, or housing built through a local housing trust.

### 2.5 Research ethics and approvals

This study was approved by University of Denver’s Institutional Review Board (Protocol #1788878-1). Our approved IRB protocol included the recruitment, data collection, and data analysis stages, as well as dissemination stage where service providers and community members, including participants, gave the team feedback regarding the policy recommendations. Signed consent forms, interview audio files, and interview transcripts were stored in a HIPAA-compliant data management system hosted at the University of Denver (REDCap) (28). A teach-back consent strategy (29) was used during the informed consent process to verify participant comprehension of the study purpose and procedures, and participants had the opportunity to ask questions before consenting to participate. To comply with COVID-19 public health guidance at the time, the interviewer wore a face mask, remained at six feet of social distance from the participant, and interviews took place outside whenever weather and the participants’ mobility permitted.

### 2.6 Data collection instrument and methods

The interview guide was developed to explore participants’ experiences with pet ownership, housing costs, and what pet policies, if any, existed at their affordable housing property (Appendix A). The interview guide also included questions about participants’ experiences finding affordable housing, feelings toward their current place of residence, experiences with other pets and people therein, and what could be improved for pets and their people in their community. The same interview guide was used across all interviews, with no changes to the questions over the course of the study.

Participants who met the eligibility criteria were informed that members of the research team would be in Houston from December 7–9, 2021, should they prefer an in-person interview. The team carried forth additional recruitment efforts during that time, including visiting nearly 30 community organizations and affordable housing properties. In total, 24 in-depth, semi-structured interviews were conducted, with 5 interviews conducted in-person during the site visit and 19 interviews conducted remotely, via Zoom video conferencing or by phone between December 2021 and February 2022. Each interview lasted between 20 and 40 min. Demographic information was collected at the end of the interview. Participants received a $50 Visa gift card.

### 2.7 Participant demographics

Twenty-four affordable housing tenants participated in this study. The majority of the participants identified as female, were aged 44–60 years, identified as Black, and were on disability or government assistance. Most participants had a high school degree or equivalent. Many participants expressed having experienced homelessness in the past and/or having lived in several different types of affordable housing over their lifetime. Seventeen participants lived with a pet; of which, six had Emotional Support Animals (ESAs). The remaining participants either previously had pets or wished to someday have a pet. A detailed summary of participant demographics is presented in Table 1.

**Table 1:** Demographics of the sample (*N* = 24).

Participant demographics		Frequency	Percentage
Sex	Female	17	70.8%
	Male	7	29.2%
Age	18–30 years old	3	12.5%
	31–45 years old	6	25%
	46–60 years old	8	33.3%
	Greater than 60 years old	7	29.2%
Race and Ethnicity	White	5	20.9%
	Black	14	58.3%
	Latino/a/e	2	8.3%
	Multi-Ethnic	2	8.3%
	Prefer not to answer	1	4.2%
Employment status	Employed Full-Time	6	25%
	Employed Part-Time	4	16.7%
	Disability or Other Government Assistance	6	25%
	Unemployed	5	20.8%
	Retired	2	8.3%
	Contract worker	1	4.2%
Highest Level of Education Completed	Less than a high school degree	4	16.7%
	High School Degree or Equivalent	10	41.7%
	Some College	5	20.8%
	Bachelor’s Degree of Higher	5	20.8%

### 2.8 Data processing and analysis

Interviews were audio-recorded using an audio recording device. The audio files were transferred from the recording devices for storage and stored in a password protected database. The audio recordings were sent to a transcription company (30) for initial transcription. To ensure the integrity and accuracy of the transcript, two members of the research team reviewed each transcript while listening to the recording and made corrections if there were any discrepancies. The transcripts were stored in a password protected database. As a final stage of processing, the transcripts were deidentified and assigned alphabetical identifiers for analysis. The participants are referred to by these alphabetical identifiers in the findings.

Analysis was conducted using the NVivo Software (QSR International, Inc.) and involved reading and re-reading transcripts line-by-line (31). Then, researchers broke down the text, mapping data into initial codes by identifying recurring patterns, phrases, and concepts. Related codes were then grouped into themes and subthemes. A deductive approach was used for the themes, keeping the general structure of the interview guide in mind (experiences finding and living in affordable housing as well as reflections on what was working well and what could be improved). Wherever possible, participants’ own words were then used for the subthemes. Throughout, researchers met regularly to engage collective reflexivity (20).

### 2.9 Trustworthiness

Consistent with recommendations for increasing the trustworthiness of qualitative research by O’Brien et al. (32), our final step was member checking. A summary of the themes and subthemes was emailed to participants, who had the opportunity to provide feedback and ensure the researchers’ analysis had accurately preserved the nuance of their lived experience. The research team also organized an in-person townhall community meeting on Saturday, June 11th, 2022 to share the study findings. Participants and community organizations that helped with recruitment efforts, as well as key stakeholdersfrom animal welfare and human social services organizations were invited to attend. The research team provided food, transportation, and childcare to attendees, as well as pet supplies and “know your rights” resources for tenants. Additional insights shared via email and at the town hall meeting were considered and integrated into the policy recommendations as appropriate.

## 3 Results

### 3.1 Barriers to finding affordable housing

There are many barriers to finding affordable housing. Participants described how difficult it is to find affordable housing in general, regardless of whether the property allows pets (“only option I had”); how important it is to find a place that allows pets, even though many affordable housing properties do not allow them (“I need to bring [my pet],” and “like choosing between life and death”); how costly pet fees, pet rents, and pet deposits can be (“if you have enough money, you can have [a pet]”); how dogs of certain sizes and breeds are more welcomed than others (“not my kind of dog”); and confusion surrounding pet policies (“must be a case-by-case basis”).

#### 3.1.1 Only option I had

Numerous participants described waitlists as one of the primary barriers to finding affordable housing. Participant E, who waited 6 years on the waitlist stated, “when I got a call to move here, this was like the only option I had.” Those with a record of any kind (e.g., criminal) also felt particularly disadvantaged when trying to find affordable housing. For example, Participant O mentioned how he was impacted by the eligibility criteria used by property managers, “I had some felonies and had difficulty finding an apartment at Section 8.” In parallel, Participant A noted “Well, my barriers are legal… I barely scrape by with my background in this place. Even though it’s been 10 years, you know.” Later in the interview, she continued to say,

“For me, it’s really more about my background that stresses me. That, you know, that I don’t care how long and how far back it’s, you know, there’s still that box and that’s discrimination too.”

Due to the difficulties in finding affordable housing in general, those without pets at the time of their search were less preoccupied with finding a unit that accepts pets. As Participant A exemplified: “When I got this housing, I did not have a pet. And I was just more concerned, after experiencing homelessness, of just being housed…I was just glad they housed humans [laughs] and, you know, left it at that.” Similarly, Participant H expressed that “the priority at the time was getting away from where I was, you know?…The priority was finding somewhere I can afford. It wasn’t - a pet wasn’t even in that equation.”

#### 3.1.2 I need to bring my pet

Those who had pets when searching for affordable housing stated it was very important to find a place that allowed their pet. Participant X reflected: “I was praying that I could get the apartment with the cat, and I was on board after she told me yes.” Participant W stated “I need to bring [my pet], I do not have nobody else to take care of him for me. It was really important for me to find [some]where he can live with us.” Despite this importance, many shared just how difficult it had been to find a place that allows pets, highlighting in particular how properties often have no-pet policies. Participant T, who has a housing choice voucher, shared:

“Most of the time if you own a pet, you're automatically almost out of compliance. It was very hard to find housing with a pet, where you can actually own a pet.”

Many participants expressed that if faced with having to choose between their pet and their housing, they would prioritize their pet. Participant R stated, for instance: “I would not stay in a place unless they allow me to have a pet. I would not. I would take my best option on a place that would allow pets.” Similarly, Participant D emphasized “I can find other places. I can sleep on the street, but I’m not giving up my dog.” For many participants, this choice was merely hypothetical and not something they have had to do in their lives to date. However, some tenants reflected on their personal experiences with this exact scenario.

#### 3.1.3 Like choosing between life and death

Some participants had experienced homelessness because they could not find an affordable housing unit that allowed pets and they refused to get rid of their companion animal. Others were forced to give up their pets to find housing. Participant G recalled their experience: “When I left [my dog] behind, you know, that was a heartbreaking thing. I had to choose if I had to leave him or I had to go out-- It’s- it’s like choosing between life and death.” Participant U explained their previous situation as, “I had a cat, and the place where I was trying to move to, they did not allow pets, with or without a deposit. Therefore, I had to give my cat to some friends, and he passed away from being—I guess mourned—he probably mourned because they reported that he would not eat, he would not drink. Even as he was leaving, he looked at me like, why? It was very stressful.” Those who were forced to give up their pets to secure housing did not want to, but felt like it was the only option they had at the time.

#### 3.1.4 If you have enough money, you can [have a pet]

Some participants believed affordable housing properties are less likely to allow pets than market-rate rental properties. Specifically, Participant H stated, “the challenge is finding housing [that is affordable AND] that accepts pets, that’s the challenge.” Participant A explained in more detail why she believes affordable housing properties do not allow pets:

“It's the affordable housing you see, because if you have enough money, you can, you know, those apartments allow pets, have pet parks in the apartment, you know. But the affordable housing where they don't treat people with respect and dignity a lot of times…Or if they-- you know, as if I have the ability to live a self-directed life, uh, you know, can't be trusted to have-- you know, “you'll ruin my apartment with your pet”, stuff like that, you know?”

Another identified barrier was the costs of pet rent, fees, and deposits. Participant U, who was trying to find a new affordable housing unit, expressed “Anywhere I live, I have to have permission to keep them without having to pay those high fees or you have to pay a pet deposit. You have to pay so much every month.” Regarding pet rent, the challenge for Participant W was “I can pay maybe once, the $300, but then they want us to keep paying monthly rent. If the dog keeps getting big, some housing, they tell you, oh, the pet rent is going to get a little bit more expensive. The dog is bigger or stuff like that.” For Participant S, the pet fees and deposit prevented her from obtaining a pet,

“A person could get a dog, but with the way that pet fees are now today and the deposits, that's just too high, it's just way too high. I would love to have a dog. I would love to have a dog, but only if the pet fees were not so high.”

#### 3.1.5 Not my kind of dog

Many tenants voiced that it is quite common for affordable housing properties to have a strong preference for small dogs. Participant R shared “you’ll find a place that you’d really like, and then they will not allow pets, or they’ll allow pets but only under 30 pounds, and not my kind of dog. You cannot have a [pitbull] hardly anywhere anymore.” In regards to how many properties accept pitbull type dogs, Participant B guessed: “I say at about a hundred apartments, maybe only two would allow.” She proceeded to say, “I was looking for an apartment for about 4 months before I was even able to find a couple that were […] allowing pitbulls to stay there. So it was just, it was a long process.” Owners of pit bull type dogs regularly felt like they were at an added disadvantage in finding housing because of their dog’s breed. For example, Participant B was approved at a property, but the property management wanted to meet the dog prior to her moving in. She shared that when she brought her dog to the property, “They’re like, ‘Oh, you can, I mean, you are approved but you have a pit bull. So I mean, you get rid of the pit bull, you can move in’.” The uncertainty around the breed restrictions at properties made it difficult for this particular participant to search for housing. Another barrier in finding affordable housing that accommodates pets was a lack of clear and upfront communication of pet policies by apartment complexes.

#### 3.1.6 Must be a case-by-case basis

Several participants stated that they did not know if their property accepted pets until it came time to sign their lease or sometimes even after they had moved in. When Participant M was asked what pet policies existed at their place of residence, they retrieved their lease during the interview, only to discover that pets were in fact not permitted: “Here we go, here we go, okay, okay. I see something on pets, here…And, it says on here that they are not authorized. [pause] But they are here…So, it must be a case-by-case basis.” Such ambiguity creates difficulty for tenants in knowing their rights regarding pet ownership.

### 3.2 Experiences living in affordable housing

Participants’ experiences living in affordable housing ranged from “I’m very comfortable” to “could be worse.” Safety concerns were commonplace, with people feeling “not protected enough” and keeping to themselves (“I do not mingle like that”). Pets provided a sense of safety and protection for their owners, for those who could have them in their units. Landlord-tenant relations also ranged from being unremarkable (“they do not mind”) to negative (“still trying to charge us more” and “threatened to kick me out or to fine me”). Given how difficult it had been to find a place, some participants reported worrying over what they would do, if ever needing to move (“do not know where (else) to go”). Having their pet designated as an ESA made tenants feel safe and secure, as they knew they could not be refused, evicted, or otherwise charged extra.

#### 3.2.1 Housing acceptability

As individuals living in affordable housing, some of whom had previously experienced homelessness, tenants felt they had little or no choice about where they lived and considering their alternatives, could not prioritize pet-keeping; worse, in some circumstances, there was a belief that this might be the best living circumstance they could ever have, leaving tenants who desired to have a pet with the belief they may never be in a position to do so.

##### 3.2.1.1 I’m very comfortable

Few participants expressed being satisfied with their current housing because they feel comfortable in their living space and like the neighborhood they live in. For example, Participant H articulated,

“I mean, you know, I was pleasantly surprised when I looked at the inside of this place before I moved in because the carpet was fresh, the walls were fresh. You know they had plenty of outlets, plenty of counter space, the appliances were nice.”

Participant H recalled how finding her place “was a godsend because it fit my needs at the time,” comparing this location to adjacent neighborhoods: “It’s quiet, you know, we have regular police patrol. We have a park that families go to, But on the outskirts of this neighborhood is third ward […] where all the shooting and gangs, drug selling and all that is. You know, but at the same time, I’m like 10 min from the medical center, 10 min from every freeway.”

Participant P was satisfied but missed her pet: “I like my current housing. I just wish I could have my [dog] with me.”

##### 3.2.1.2 Could be worse

Some participants were overall ambivalent, as illustrated by Participant M: “As a whole, could be worse, so it’s okay… It’s okay, right? As a whole for what it offers.” Others noted complaints about cleanliness (“Despite the roaches and things, but we are solving these problems with new management,” (Participant W)) and housing design, often directly related to their unit being too small. Participant Q, who lives in a single room occupancy (SRO), described their apartment as being unsuitable: “real condensed and not enough room for the pet to move around.” A resident of a different SRO property (Participant O) similarly stated,

“It's not the best place in the world to live, but we're a bunch of disabled vets. We're all old and they're carrying us out one at a time… ‘Don't let this be my last stop,’ kind of makes you want to get out of here, but it's all right. The apartments are small. It's an old hotel is what it is and they just converted the hotel rooms…I would like to have a regular-sized apartment. That would be nice.”

#### 3.2.2 Housing safety

Some tenants indicated they had concerns related to safety in their housing, which resulted in fear (particularly of losing personal possessions) and anxiety (around personal safety, or someone harming their pet to get at them). Because of concerns related to safety at their housing, tenants discussed self-protective behaviors like keeping to themselves, which may have impacted the extent to which having pets resulted in the development of meaningful friendships or valuable social networks.

##### 3.2.2.1 Not protected enough

Safety concerns were a common issue for many tenants. As Participant F shared: “Um, basically, sometimes I feel not protected enough because we did have a couple of incidents happen on this property. And by me being a woman of young age, it’s very scary to live alone cause you never know what may happen.” Similarly, Participant U disclosed,

“Oh, we had break-ins, cars stolen. A couple of the gates that they have around here have been ran over or torn up. They have all kinds of traffic coming in and out, people that don't really live here….they're not very secure right now. I don't know if they're planning on doing anything about it because I've been here about seven years and used to have a security guard that used to walk through, but they don't have that anymore.”

##### 3.2.2.2 I do not mingle like that

At times, participants described neighbor conflicts related to pets, citing that some people do not like animals or may be fearful and that some animals are aggressive. Participant K shared about a negative encounter with one of his neighbors,

"Today, I was sitting outside my door. See, my dog was between my legs minding her business, ain't saying nothing, and this idiot walk up the door. He's like, ‘Where is she at? I'm gonna kick her.’ I said, ‘Homeboy, my dog…You bet you ain't gonna kick this dog.’ I mean, why would you even say that? My dog isn't bothering you. So that's self-explanatory, he don't like the dog, right?"

Given this potential for conflict, some participants talked about keeping to themselves to avoid issues. For example, Participant N stated, “All I worry about is me and my pet, everybody has got their pet, I let them worry about their pet. I do not mingle like that.”

To combat feelings around safety concerns, several participants spoke to how pets provide a source of protection. As Participant T said, “Well, first off, my dog that I did have, a family pet, he’s more of a guard dog. I do not like guns like that. He would bark very loudly when someone would come up to the door. He’s served as extra security in various situations.” Similarly, Participant W voiced, “…sometimes people want a dog or need a dog to stay safe because you’ll never know if somebody’s going to decide to come in your house and a dog is a protector, they protect their owners. I really do think that it’s important for housing to accept dogs.” Additionally, Participant J shared, “when [my dog] hears knocks at the door or something she growls and she- she’s very alert and attentive.” Participant T shared the vital role pets play when people may be living in less-than-ideal circumstances: “The worse the neighborhood, the more the need for the pet. The worse my mental state is, the more the need for a pet. It’s on a spectrum if you will.”

#### 3.2.3 Landlord-tenant relations

##### 3.2.3.1 They do not mind

When it came to landlord-tenant relations regarding pets, most participants claimed to have unremarkable encounters with their landlord, with many equating no problems as positive experiences. As Participant V shared, “They do not mind [my dog] as long as I clean up after him, they do not mind.”

##### 3.2.3.2 Still trying to charge us more

Some tenants had negative experiences with their landlord. These included being charged more money than the agreed pet deposit, feeling distrustful that the pet deposit would be returned at the end of tenancy, and being afraid to ask their landlord about getting a pet. For example, Participant W, shared:

“We had that issue a couple of years ago, we had another dog and they didn't let my dogs be on the balcony. They had to be inside. I had a newborn baby, so I couldn't really have them inside all the time… I tried to reason with some of the landlords explaining to them that my dog wasn't going to be inside all the time…We take them out. My husband takes him out for walks and he cleans up after her, but they weren't trying to hear it. They didn't care…We had to move from there, and then we had another issue where at the same apartment complex where we paid the fee of the dog and they were still trying to charge us more fees after that.”

##### 3.2.3.3 Threatened to kick me out or to fine me

The most severe scenario was when Participant T was keeping a pet at their home without the landlord’s consent, “…they put a notice on my door that said, ‘If you do not get rid of this pet, there’s going to be some type of negative retaliation.’ I cannot remember if they threatened to kick me out or to fine me.” To avoid disciplinary action, Participant T returned the dog to the person they purchased them from. Participants worried over receiving complaints about the pet.

#### 3.2.4 Housing (in)security

##### 3.2.4.1 Cannot make you get rid of your dog

While some participants worried about getting evicted, those with ESAs experienced a sense of housing security, knowing housing providers could not deny, refuse, charge extra, or discriminate against them. As Participant K said, “being an emotional support animal because they cannot make you get rid of your dog.” Participant L expressed confidently that: “As long as I have their immunizations and my letter from the doctor, I have no problem or have any fear of being put out.” Additionally, Participant O shared they would not feel at a disadvantage when looking for housing with their ESA because:

“They said an emotional support animal is not considered a pet and that they can't be refused. They can't charge you a deposit.”

##### 3.2.4.2 Do not know where [else] to go

Some tenants felt stable in their current housing, while others discussed concerns around stability. Many participants mentioned experiencing homelessness in their past and having to move around a lot, and a number of participants talked about how they currently needed to or would like to move. For example, Participant C shared how his family and their neighbors are experiencing housing instability, highlighting how their current housing “is supposed to be getting torn down…they are supposed to be building a big freeway right here. Right now, they are giving out vouchers for us to find somewhere else to go. It is kinda challenging because right now we do not know where [else] to go.” Additional reasons for wanting or needing to move included feeling unsafe, not being able to afford the property they live at, not having enough space at their unit, or worrying about finding a place that would allow them to keep their pet. Participant H elaborated on this:

“Where I can afford to live doesn't allow pets, so right now I can't have a pet but it's like eating me inside because I'm like, okay, I need more. I'm trying to find a night job or weekend job so that I can save money so that I can move somewhere where I can have a pet. Like, I have to make it work.”

#### 3.2.5 What is working well and opportunities for improvement

Based on these lived experiences, participants were invited to share what is working well and what could be improved when it comes to pets and affordable housing.

##### 3.2.5.1 Benefits of having affordable housing that accepts pets

Tenants identified numerous personal benefits to having affordable housing that accepts pets. Many talked about how pets are like family members, they make people happy, provide motivation, and get people up and moving. Participant L shared about the emotional benefits of pets in their comment,

“With the pandemic, you know, it's a lot of times you couldn't go anywhere, you couldn't do anything, but if you had your little fur babies there, you didn't have to worry about, you know, being lonely. You know, they keep me from being lonely, that [my dog] is very entertaining. And [chuckles] so I just feel like that they're a very important part of-- especially if you're missing something in your life, I feel like that they can help fill a big void in your life if that's what you want them to do.”

Pets were also described as a way for neighbors to build connections: “The positive side of it I guess would be, I think dogs and animals, in general, make people- you get to meet people that way. People go out walking their dog, everyone wants to pet it.” (Participant R). Lastly, tenants highlighted that when housing accepts pets, it helps prevent animals from being homeless. Participant B spoke directly to this idea, “we could be in a situation where nobody accepts dogs, and we-- there’s, like, abundant amount of more strays and more dogs and shelters. So, I feel like apartments and houses that accept [pets] ensure that [not only people but] dogs have a home as well.”

##### 3.2.5.2 One bad apple ruins the whole bunch

Despite these benefits, participants also provided reasoning to why there are negative impacts of having housing that allows pets. One of the negative impacts was what some participants described as “irresponsible pet owners.” Participant T illustrated this as:

“Well, some people don't pick up after their pets. Some people neglect their pets. If you’re letting your cats poop or your dogs poop and pee all over the house, it attracts bugs and everything. That's the negative impact. Everyone doesn't take the same amount of care to their pets as other people do.”

Participant R explained an irresponsible pet owner as, “You got people that let their dogs run around, that are violent animals without a leash, scaring kids and things, or biting people.” Several participants elaborated on how the pet policies are likely informed by rare issues (exception not the norm). For example, Participant T stated, “I understand why it’s hard because you know what they say, “one bad apple ruins the whole bunch. It’s one of those types of things” Similarly, Participant S relayed, “If one person does something bad, it falls back on everybody else to make it bad for everyone else.” As such, participants did not necessarily believe the pet policies are fair for everyone.

##### 3.2.5.3 Appreciation for leash laws in common areas

Most of the affordable housing properties had leash policies, and this was something that participants generally stated they liked. Specifically, Participant S said, “the leash laws, we still have them, dogs must be on leashes, which is good.” Participant B provided reasoning as to why she liked the leash policies by saying if pets were not on a leash, “I feel like it could cause unnecessary accidents.”

##### 3.2.5.4 Need to consider pets in design of housing and neighborhoods

Although people liked the leash policies, many participants also talked about wanting a place for their pet to be off leash. For example, when asked what could be better for pets and pet owners at the property, Participant V shared, “Probably a more designated area on the property to take care of their business. I’m trying to find something better than a plastic bag to pick up his stuff. It could be a better-designated area where we can let them loose and they can explore instead of being on the leash all the time.” Similarly, Participant B expressed, “Because like if I was to walk my dog, like there’s playgrounds and there’s children and there’s kids and there’s people running around. So, I feel like they do not give an area for the owner to have peace of mind while walking their dog.” The majority of participants expressed that there was a lack of accommodations or amenities for pets within affordable housing properties, with desired accommodations being pet parks, poop bag dispensers around the property, and green spaces to walk pets.

## 4 Discussion

The goal of this study was to document the lived experiences of affordable housing tenants in Houston, TX, with a focus on pet ownership. Twenty-four participants shared their experiences related to three overarching themes: barriers to finding and maintaining pet-inclusive affordable housing, experiences living in affordable housing related to pet ownership, and what tenants believed was working well and where there were opportunities for improvements related to pet-keeping in affordable housing. We discuss these findings in detail below.

We found that tenants’ ability to find housing that accepts pets was compounded by difficulties in securing affordable housing in general, including an overall shortage of available units, limited suitable housing options, long waitlists, and eligibility criteria. These experiences are likely a result of the historical and ongoing disinvestment of affordable housing in Houston overall, exemplified by the demolition and deterioration of public housing, displacement of low-income families due to gentrification, racial segregation, and an increasing number of individuals who are rent burdened (33). Some tenants in our study reported feeling unsatisfied with where they lived, primarily due to complaints about housing design or safety concerns. Present pet owners worried over housing instability and where else to go if they ever needed to move; however, such concerns were less common if their pet was designated as an ESA. Notably, several tenants in this study indicated that, because of safety concerns at their housing unrelated to pets, they did not experience the social benefits of pet ownership often cited in other human-animal interaction studies [e.g., (34)]. This indicates that placement of affordable housing in areas of high crime may be preventing pet-owning tenants from experiencing the myriad benefits related to increased social interaction and cohesion often found in pet-friendly communities.

This study found that ESAs provided affordable housing tenants a sense of housing security, because under the Fair Housing Act, ESAs are protected as a “reasonable accommodation” in housing and shelter (35–37). However, there have been several efforts to overturn or limit the use of ESA designations. For example, California passed a law in 2022 requiring a therapist and client to have a 30-day relationship before the therapist is allowed to write an ESA letter (38). While this may seem an innocuous change, a 30-day waiting period may have significant implications for people who are needing to enter housing under short notice, including individuals leaving situations of domestic violence or those entering housing after being unhoused. Ultimately, laws such as this will most negatively impact the most vulnerable renters with the least resources. Furthermore, all companion animals can arguably provide comfort to their owners [e.g., (39)] and requiring only marginalized pet owners to “prove” their animal is of their emotional benefit is yet another systemic inequality in access to pet ownership and the human-animal bond (40).

This study furthermore documents how challenging and traumatic it is for people to give up pets to secure housing. Housing is a top reason for companion animal relinquishment to animal shelters (8, 9, 41), but none of the participants in our study reported giving their pet up to an animal shelter when faced with such a dilemma. Instead, participants sought alternative options, such as giving their pet to a friend or family member, returning their pet to where they got them from initially, or leaving them under the care of the next occupant of their unit. Emerging strategies to help keep people and pets together when they are facing housing insecurity include co-sheltering efforts, emergency boarding, and temporary foster programs (42). More research is needed to understand the scope of how housing issues are disrupting the human-animal bond in communities to better establish evidence-based programs.

Aspiring pet owners in this study discussed the ways that policies in affordable housing may be restricting people from obtaining a pet and thus denying them the potential health benefits of the human-animal bond. Zimolag and Krupa (16) found that the two most common reasons for not owning a pet among individuals who wanted one were cost and the landlord not allowing pets. Aspiring pet owners in our study cited similar barriers, including difficulty finding affordable housing that accepts pets and costly pet fees. Specifically, they shared the sentiment that “if you can pay, you can [have a pet],” highlighting how affordable housing residents feel disproportionately impacted by surcharges. Future research should examine more closely how bans, restrictions, and surcharges impact the ability for people to have pets.

Nearly 40 years ago, the U.S. Congress passed a law prohibiting federally-assisted housing providers for the “elderly or handicapped” from disallowing tenants to keep “common household pets” in their dwellings (43). The law not only prohibited a housing provider from discriminating against tenants with pets during the tenant selection process, but ensured that a tenant could not be denied continued occupancy based on the presence of a pet. In practice, this law has been less effective in increasing access to pet-friendly housing because it expressly allows for “reasonable rules for the keeping of pets,” including pet size and type of pet (43). The Department of Housing and Urban Development (HUD) regulations go so far as to allow property owners and managers to have different pet rules *within individual projects*, which opens the door for discrimination against individual tenants and pets and makes it challenging for potential tenants to understand the applicable pet rules at any given property. These inconsistencies and opportunities for discrimination were reflected in our interviews: our study found that pet policies appear to be applied on a case-by-case basis, rather than having a consistent, written policy applicable to every tenant. In addition, for opportunities for discrimination, this makes it difficult for tenants to understand the rules and expectations for keeping a pet in their home. This study also found that in affordable housing, providers fail to adequately advertise pet policies and that it can be difficult for tenants to understand their rights. Broader recognition of pets in housing as a tenant’s rights issue has the potential to inform more equitable housing solutions for both people and pets.

In light of these findings and, to better support people and their pets, we must recognize the issue of pets in affordable housing as a systemic, One Health/One Welfare issue (44). A One Health/One Welfare approach would see animal welfare advocates working alongside housing advocates and other human social services to create a comprehensive approach to addressing housing needs and resource deserts as both a human and animal welfare issue (45, 46). Often, advocates and practitioners (e.g., mental health professionals, animal shelters) operate at an individual level, resolving issues for individuals and their pets one case at a time. We argue that, while important to help the person and pet in front of you, we must also work toward systemic interventions and policy change to address the root causes of the issues for broader and more sustainable impact.

### 4.1 Recommendations for policy and practice

Based on these findings, and drawing on policy efforts to date, we recommend the following to respect people’s rights to live with companion animals regardless of income:

#### 4.1.1 Remove “blanket pet bans” in all housing receiving federal, state, or local funding

Requiring all housing receiving federal, state, or local funds to allow pets would mitigate at least one challenge facing low-income tenants when they are searching for an affordable home. Because of racial inequities in rates of homeownership in the U.S., policies targeting subsidized housing will likely benefit Black and Latino/x/e people who are most at-risk of housing insecurity (47). Furthermore, removing “blanket pet bans” from housing policy could help eliminate the need for differentiation between ESAs and companion animals, given that any pet can arguably provide support to their owners (48). We suggest that advocates aiming to address ESA misuse should shift their focus from punitive laws to advocating for pet-inclusive housing policies, thus eliminating the added hurdle of vulnerable tenants needing to prove the benefits their pets bring, in order to keep them, and alleviating ethical concerns for mental health professionals who are hoping to assist pet-owning clients in securing housing (49).

#### 4.1.2 Remove surcharges for pet ownership in housing and breed/size restrictions

We suggest that efforts to prescribe pet policies in affordable housing would be most effective if they prohibited non-refundable fees and rents and removed restrictions based on breed or size. In the U.S., there has only been one successful effort at the state level to ban or cap pet rent in market-rate housing (50), although several states, including Texas (51, 52), have attempted to pass such laws in recent years. Washington recently introduced legislation capping pet rent and banning pet-related nonrefundable fees, however the bill failed to pass in 2024 (53). Further, no federal regulations exist in the U.S. regarding pet fees, pet deposits, or pet rent, meaning there is no legal guidance regarding what is considered a reasonable or acceptable surcharge for pets in housing.

#### 4.1.3 Require written and consistent pet policies at each property

We suggest that affordable housing funded through federal, state, or local programs be required to submit written pet policies to the enforcing agency. This policy recommendation is in line with existing reporting requirements for most, if not all, affordable housing properties, which must submit policies such as tenant selection criteria and other written policies and rules to which tenants must adhere pursuant to their lease.

#### 4.1.4 Pet policies should be publicly available (on a website) or easily accessible to potential tenants

We suggest that housing agencies should create and keep updated a list of currently “pet-friendly” housing developments, including their pet policies, available on their website. Making this information easily accessible, along with the various other metrics already collected and shared on these agencies’ websites, would give tenants the full picture of what is required at each property and remove a significant barrier during their search process. Importantly, this is also something that any agency could do immediately, without the typically lengthy process to change the details of pet policy requirements through law-making processes.

#### 4.1.5 Improve experiences of tenants with pets

As a result of historic racist decision-making at multiple levels of government, the majority of Houston’s affordable housing was built in neighborhoods with high poverty rates, low-quality schools, high crime rates, poor employment prospects, high rates of exposure to environmental pollutants, and a lack of public infrastructure and resources, including healthy foods, green spaces, health care, veterinarians, pet grooming services, and pet stores (26, 54). These interviews indicate that affordable housing placement has significant impacts on how acceptable and safe tenants perceive their housing and appears to impact how tenants are able to care for, and benefit from, their pets, if they are allowed to have a pet at all. Where available, development of new affordable housing and rehabilitation of existing affordable housing should be placed in areas of deconcentrated poverty, close to dog-friendly green spaces and within reasonable walking distance of stores providing pet-related supplies (e.g., full-service grocery stores or pet stores). All housing receiving state or local funding should also provide dog waste bags and bins at no additional cost. Considering the needs of pet owners in development locations and providing these amenities and resources may mitigate some of the reported negative experiences with pets in housing as noted in several participants’ responses. For example, tenants may be less inclined to let their pet off-leash in violation of pet policies if there is a designated off-leash area and may be more likely to pick up after their pet if there are bags and bins readily available. These changes would serve to keep pets and people safer and may reduce neighbor conflict.

## 5 Strengths and limitations

There are several strengths of this study that we highlight here. First, little research in this area has captured the firsthand experiences of underrepresented groups in human-animal interaction research; our sample was predominantly made up of individuals from minoritized racial backgrounds, and who were low-income and struggled with housing insecurity. Furthermore, much of the research in this area captures those who interact with the animal shelter system (i.e., current pet owners and those who are relinquishing to the shelter); our sample includes individuals who had not interacted with the shelters when rehoming their pets, as well as those who were former and aspiring pet owners. Including the voices of these underrepresented and marginalized groups is an important contribution to the empirical literature and can inform policy decisions that impact vulnerable groups beyond what previous research has shown.

Our study is not without limitations. First, this study was conducted solely in the City of Houston and thus may not be generalizable to other cities. Given the differences in affordable housing policies across the U.S., more research is needed to understand the similarities and differences between residents of Houston and other areas. Seventeen of the 24 participants currently kept pets, which could bias the results to over-represent those who were able to keep pets and under-represent those who experienced barriers to pet ownership that could not be overcome. Additionally, this study did not have representation from individuals who did not speak English. This is limiting in that language proficiency is a known housing barrier and could be impacting individuals’ experiences of finding and maintaining affordable pet-inclusive housing. Furthermore, our sample did not include many individuals under 30 years old. Future research should explore these experiences among individuals who do not speak English, as well as those who are younger.

## 6 Conclusion

In recent years, there has been a significant uptick in research and policy advocacy on the impact of the housing crisis, in the U.S. and globally, on pet relinquishment and the human-animal bond. In both traditional animal welfare and affordable housing research and discourse, the lived experiences of tenants and their pets in subsidized affordable housing has been largely overlooked. This is particularly apparent in policy advocacy addressing barriers to pets in rental housing, which has largely focused on eliminating breed and size restrictions. This study provides important insight about the types of policy changes that would be most beneficial to tenants and supports expanding policy advocacy beyond traditional legislation to eliminate breed and size restrictions to include new policies improving access to important pet-related infrastructure and resources at subsidized affordable housing developments. Additionally, this study reinforced that ESAs provided a sense of housing security and calls into question efforts to increase barriers to access ESA designations as counterproductive to the Housing First approach to keeping people (and their pets) housed. However, ESA designation is yet another administrative hoop that marginalized individuals have to jump through in order to maintain the human-animal bond in the context of housing inequalities and thus does not address the root causes of the issue. Importantly, efforts to increase housing security among pet owners must be systemic in nature and address the root causes of the issues for meaningful, equitable, and sustainable change.

## Data Availability

The raw data supporting the conclusions of this article will be made available by the authors, without undue reservation.

## Appendix Interview guide

1. How important was it to you to find housing that accepts pets?
2. How does having a pet influence where you chose to live?
3. What were the challenges in finding a housing property that accepts pets?
4. Did you feel you were treated fairly by landlords in the process of finding housing? How so?
5. Did you feel like you were at a disadvantage when looking for housing because you have a pet? How so?
6. How stable or secure do you feel in your current housing? Do you foresee any challenges to staying in your current housing?
7. How does having a pet influence your ability to stay in your current place?
8. Does having a pet influence your ability to move to another place?
9. What does the word ‘pet-friendly’ mean to you? How could the place you are currently living in be better for pets and pet owners?
10. What do you think are the impacts (positive or negative) of having housing that allows pets in your community?
11. How did the process of finding housing for you and your pet(s) impact you?
12. How has the availability of housing that accepts pets in your community impacted your pet?
13. Could you tell us about a time you felt like you were forced to choose between your pet and your housing?
